# Distrust, False Cues, and Below-Chance Deception Detection Accuracy: Commentary on Stel et al. (2020) and Further Reflections on (Un)Conscious Lie Detection From the Perspective of Truth-Default Theory

**DOI:** 10.3389/fpsyg.2021.642359

**Published:** 2021-07-21

**Authors:** Timothy R. Levine

**Affiliations:** Department of Communication Studies, University of Alabama at Birmingham, Birmingham, AL, United States

**Keywords:** deception, unconscious, deception detection, truth-default theory, lying

## Introduction

This essay has two distinct aims: commentary on Stel et al. (2020) and addressing (Un)Conscious Lie Detection from the Perspective of Truth-Default Theory. Stel et al. (2020) advance the counter-intuitive claim that distrust inhibits truth detection by prompting more deliberate reliance on false beliefs about deception cues. Their proposed mechanism lacks plausibility because cues are generally non- rather than anti-diagnostic. Truth-default theory offers different predictions that may explain the inconsistent findings observed across the previous literature. When in the truth-default state, accuracy is a function of message veracity. People correctly believe honest messages and are duped by lies. In prompted demeanor-based lie detection tasks, accuracy is a function of sender demeanor-veracity matching and sender sampling. In lie detection tasks where message content is diagnostic, deliberative processing improves accuracy.

## Commentary

Stel et al. (2020, p. 1) wrote: “distrust especially hampers the detection of truth, which is partly due to more reliance on false beliefs about deception cues. These results corroborate the idea that deliberative conscious information processing may hinder truth detection, while intuitive information processing may facilitate it” (see Street and Vadillo, 2016 for a discussion of unconscious and indirect lie detection). I agree that (a) distrust hampers correct inferences about honest communication and that (b) suspicion and distrust involve conscious processing. However, I believe this has little to do with deception cues. Although Stel et al.'s findings were replicated in their second study, I am skeptical of the robustness and interpretation of their findings and conclusions because they do not align with my understanding of prior findings or my own theoretical perspective which is described in the second part of this essay.

Decades ago, when I was a graduate research assistant, my professor Steve McCornack wondered if the detrimental effects of truth-bias on accuracy could be counteracted by forewarning the possibility of deception. We found that warning people about the chances of deception reduced, but did not eliminate, truth-bias (McCornack and Levine, 1990; also see replication by Kim and Levine, 2011). Although Stel et al. (2020) did not forewarn participants, conceptually forewarning should lead to distrust as Stel et al. define it.

My understanding of our 1990 findings changed a decade later when Hee Sun Park came up with the “veracity effect” (Levine et al., 1999). Suspicion/distrust decreases accuracy for truths, increases accuracy for lies, and the gains and losses average out overall (Kim and Levine, 2011). Suspicion/distrust reduces but does not eliminate or reverse the veracity effect as Stel et al.'s 2020 findings suggest. With conventional research designs people are (a) slightly above chance accuracy overall, (b) truth-biased, and (c) more accurate at truths than lies. These findings are remarkably robust. Findings to the contrary are refuted by literally hundreds of results (Bond and DePaulo, 2006; see Levine, 2020 for an updated review).

Stel et al. (2020, p. 5) found: “when distrust was activated, participants inaccurately judged truth-tellers as more deceitful than liars (*d* = −0.89), [*d* = −2.09 in study 2]. When trust was activated, participants accurately judged liars as more deceitful than truth-tellers (*d* = 1.07).” These findings for distrust conflict with the literature. Cue-based truth-lie discrimination is slightly better than chance (about *d* = + 0.4), not one or two standard deviations below chance (Bond and DePaulo, 2006). Training participants to consciously look for wrong cues does not produce below chance accuracy (Levine et al., 2005).

Stel et al. (2020) explain their below-chance accuracy by increased reliance on false beliefs about deception cues. Their mechanism, however, cannot not explain their findings. The cues in question (gaze aversion, blinking, smiling, illustrators, hand movements, body movements, posture, and appearance) lack diagnostic value. They are not strong reverse-sign indicators that could produce a huge effect size of *d* = −2.09. My work on matched and mismatched senders shows that any given cue can be diagnostic for one sender, but not at all, or even reverse, for another (Levine et al., 2011). Across senders, however, this averages out to slightly better than zero cue diagnostic utility as data accumulate (Bond et al., 2015). Further, meta-analysis also shows that cue diagnostic utility and cue reliance are positively associated (Hartwig and Bond, 2011). Thus, increased reliance on specific cues cannot explain substantially below-chance accuracy unless the particular senders are highly idiosyncratic and atypical of the population of communicators.

## The Truth-Default Perspective on Conscious and Less Conscious Lie Detection

Do trust, distrust, or suspicion facilitate deception detection accuracy? According to truth-default theory (TDT, Levine, 2020), this entirely depends on if the communication is honest or deceptive. Trust is highly advantageous in environments were honesty predominates and where the cost of being duped is not harmful. Fortunately, this characterizes the vast majority of human communication situations where deception is much less probable than it is in the lab. Suspicion is helpful in situations where deception is pervasive or pernicious. The trick is being selectively suspicious or distrustful in the right situations and applying sound critical thinking skills to communication content understood in context.

Once researchers directly ask participants about trust, honesty, suspicion, or deception, as in the Stel et al. (2020) experiments, issues of honesty and deception are brought to mind. TDT proposes that absent this methodology-induced priming, deception often does not come to mind and accuracy at detecting lies in real time approaches zero (Levine et al., 2020). The human unconscious is a believer. Suspicion, doubt, and distrust require activation and cognitive effort.

TDT draws distinctions between cues, demeanor, and communication content as inputs for making veracity assessments (Levine, 2020). Cues are specific behaviors such as gaze aversion, hand movements, or the number of details in a statement. Two insights from TDT are (a) cues do not travel alone, and (b) the impact of cues on impressions is collective. Most deception research, in contrast, treats cues as if they are statistically independent from other cues. This is not how cues function. Demeanor refers to constellations of inter-correlated cues that are given off and perceived as a package or perceptual whole. Impressions about honesty or deceptiveness are based on overall demeanor more than specific cues. The particular diagnostic value of any specific cue is diluted in human detection tasks because judgments, especially intuitive judgments, are strongly impacted by a person's overall demeanor.

When looking at demeanor-based veracity judgments across large numbers of senders and judges, systematic patterns in errors become evident. Across judges, judgments of individual senders tend to converge producing predictable trends in correct and erroneous veracity assessments. Some senders are transparent across judges. Almost everyone gets them right. I call these senders “matched” because their demeanor matches their veracity. There is also a sizable group of senders who are mismatched or exhibit negative transparency. These folks are smooth and seamless liars as well as awkward, socially-unskilled honest people. They systematically produce erroneous impressions.

Reliance on demeanor pushes accuracy down toward chance because both individual cues and honest demeanor lack robust diagnostic value. As previously mentioned, cue reliance and cue diagnostic value are positively correlated (Hartwig and Bond, 2011).

Moreover, cues and demeanor are not how people detect lies outside the lab (Park et al., 2002). When people can actively assess plausibility and motives, fact-check statements, have relevant prior knowledge, and can encourage honesty, conscious and deliberate lie detection is much better than lie detection experiments suggest (Levine, 2015). That is, if people are allowed to detect deception the ways they do in their everyday lives, conscious and deliberate cognitive processing facilitates lie detection. Regardless, incorrect folk beliefs about non- or low diagnostic indicators cannot produce or explain below-chance performance. That requires a cue that is massively anti-diagnostic or a set of negative-transparency (mismatched) senders who are not representative of the population of senders.

TDT makes the following set of predictions regarding intuitive and mindful processing of honest and deceptive communications. If there is no prompting at all and people are doing communication business as usual, then people simply believe regardless of communication veracity. The truth-default prevails. In order for the possibility of deception to come to mind, there must be some prompting or “trigger.” For example, maybe a research method requires the subject to rate honesty or deception on a scale. But absent this or some other trigger, communication is passively accepted at face value. If that communication is a lie, the target of the communication is duped.

My favorite example of this is the use of identity deception in research using research confederates (research assistants posing as other subjects). Those of us who use research confederates know perfectly well that real research subjects almost never suspect that the confederates are imposters. Accuracy is near zero, not 54%.

Once prompted to make a judgment, then the more intuitive the judgment, the more it is demeanor based. If this is correct, then the advantage of intuitive (versus mindful) judgments depends on the demeanor of person being evaluated. The greater the proportion of matched senders, the more advantageous intuitive processing. However, intuitive processing would be counterproductive with mismatched senders. That is, the advantage of intuitive processing is a function of the proportion of matched and mismatched senders being judged. This presumes that humans process demeanor intuitively.

This might explain the mixed findings for processing type in the literature. The advantage goes to intuitive processing when senders tilt toward matched, there is no effect when matched and mismatched balance out, and the findings reverse when the senders tilt mismatched. It would take a large sample or senders or chance for matched and mismatched senders to average out. Most deception detection studies, including Stel et al. (2020), involve fewer senders than judges. Given this, TDT predicts widely variable results in both directions and a lack of replication across studies using different small samples of communication.

If prompted to make a judgment, the more conscious and mindful the processing, the more accuracy comes down the diagnostic value of communication content. Communication content refers to the substance of what is said. When communication content is provided with sufficient context or situation familiarity, then critical thinking becomes efficacious. One can assess plausibility and the consistency of statements with already known or discoverable facts and evidence. Conscious and mindful the processing should produce accuracy near chance when communication content lacks context and diagnostic value. As the potential diagnostic value of what is said increases, critical thinking and investigative skills become increasing useful, and detection accuracy is expected to improve concomitantly.

## Author Contributions

The author confirms being the sole contributor of this work and has approved it for publication.

## Conflict of Interest

The author declares that the research was conducted in the absence of any commercial or financial relationships that could be construed as a potential conflict of interest.
